# A draft genome provides hypotheses on drought tolerance in a keystone plant species in Western North America threatened by climate change

**DOI:** 10.1002/ece3.8245

**Published:** 2021-10-19

**Authors:** Anthony E. Melton, James Beck, Stephanie J. Galla, Jerry Jenkins, Lori Handley, Min Kim, Jane Grimwood, Jeremy Schmutz, Bryce A. Richardson, Marcelo Serpe, Stephen Novak, Sven Buerki

**Affiliations:** ^1^ Department of Biological Sciences Boise State University Boise Idaho USA; ^2^ Department of Computing Boise State University Boise Idaho USA; ^3^ HudsonAlpha Institute for Biotechnology Huntsville Alabama USA; ^4^ USDA Forest Service Rocky Mountain Research Station Moscow Idaho USA

**Keywords:** adaptation, aquaporins, drought stress, genome mining, genome‐to‐phenome, sagebrush

## Abstract

Climate change presents distinct ecological and physiological challenges to plants as extreme climate events become more common. Understanding how species have adapted to drought, especially ecologically important nonmodel organisms, will be crucial to elucidate potential biological pathways for drought adaptation and inform conservation strategies. To aid in genome‐to‐phenome research, a draft genome was assembled for a diploid individual of *Artemisia tridentata* subsp. *tridentata*, a threatened keystone shrub in western North America. While this taxon has few genetic resources available and genetic/genomics work has proven difficult due to genetic heterozygosity in the past, a draft genome was successfully assembled. Aquaporin (AQP) genes and their promoter sequences were mined from the draft genome to predict mechanisms regulating gene expression and generate hypotheses on key genes underpinning drought response. Fifty‐one AQP genes were fully assembled within the draft genome. Promoter and phylogenetic analyses revealed putative duplicates of *A. tridentata* subsp. *tridentata* AQPs which have experienced differentiation in promoter elements, potentially supporting novel biological pathways. Comparison with nondrought‐tolerant congener supports enrichments of AQP genes in this taxon during adaptation to drought stress. Differentiation of promoter elements revealed that paralogues of some genes have evolved to function in different pathways, highlighting these genes as potential candidates for future research and providing critical hypotheses for future genome‐to‐phenome work.

## Graphical abstract

A draft genome and novel mining/analysis pipeline was sued to assess Aquaporin genes in a nonmodel, ecologically significant plant species. We found that the genome of this species is enriched in Aquaporin genes and that homologs have likely diverged experienced promoter element divergence, potentially contributing to drought adaptation.

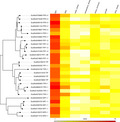

## 1 INTRODUCTION

Drought is a major factor determining plant survival, growth, and reproduction, as well as species distributions (Brenes‐Arguedas et al., 2009; Samarah, 2005). Drought stress has numerous deleterious effects on plants, including reduced growth (Samarah, 2005), increased production of reactive oxygen species (ROS; Cruz De Carvalho, 2008), and reduction of photosynthetic efficiency (Galmés et al., 2007). Water deficits generate hydraulic and chemical signals, particularly abscisic acid (ABA), that trigger signaling cascades and ABA‐dependent and ABA‐independent transcriptome changes (Chaves et al., 2003; Christmann et al., 2013; Lata et al., 2015; Takahashi et al., 2018). Plants respond to water deficit through mechanisms that reduce water loss, increase water uptake, and alter hydraulic conductivity (Sharp et al., 2004; Kaldenhoff et al., 2008; Hsu et al., 2021). In the long term, adaptations to dry environments come through the evolution of structural, biochemical, and physiological traits that maximize the ability to acquire and retain water (reviewed in Shinozaki & Yamaguchi‐Shinozaki, 2007; Shinozaki et al., 2003). These traits may include deep roots, thick cuticles, sunken stomata, CAM metabolism, and characteristics that reduce or enable repair of xylem embolism (Fahn, 1964; Lüttge, 2004; McElrone et al., 2007; Secchi et al., 2017).

Aquaporins (AQP) are a large family of proteins known to function in the transport of water and other molecules across cell membranes (Yakata et al., 2007; Azad et al., 2012; Uehlein et al., 2012; Reviewed in Li et al., 2014; Afzal et al., 2016). The defining characteristics of AQP proteins include having six alpha‐helices, including two conserved asparagine–proline–alanine (NPA) motifs (Mitsuoka et al., 1999; Murata et al., 2000). While the NPA motifs are generally highly conserved, there are some AQP genes that have undergone mutations of the alanine residue in the NPA motif (Ishibashi, 2006). AQPs in flowering plants comprise five subfamilies: (1) NOD26‐like intrinsic proteins (*NIP*s), (2) plasma membrane intrinsic proteins (*PIP*s), (3) small basic intrinsic proteins (*SIP*s), (4) tonoplast intrinsic proteins (*TIP*s), and (5) X intrinsic proteins (*XIP*s) (Danielson & Johanson, 2008). Genes from each subfamily tend to move water or other substrate depending on their NPA motifs. Some AQPs, such as *NIP*s, have acquired a mutation in their NPA motif, such as alanine to leucine, which confer the ability to move substrates such as urea or ammonium (reviewed in Chaumont et al., 2005; Kaldenhoff & Fischer, 2006; Maurel, 2007).

Most of the water plants take from the soil is lost to the atmosphere via transpiration (Steudle, 2001). In this water flow through the soil–plant–atmosphere continuum, AQPs play an important role in controlling the radial movement of water from the soil to the root xylem as well as water movement from the leaf xylem to evaporation sites in the mesophyll (Sade & Moshelion, 2017). Various environmental and internal changes such as in soil moisture, evaporative demand, salinity, and ABA can alter the expression and activity of AQPs and plant hydraulic conductivity (Afzal et al., 2016; Ding et al., 2015; Fang et al., 2019; Maurel et al., 2016). Through these changes in expression and activity, AQPs contribute to regulate plant water balance and maintain cellular water homeostasis, which ultimately affects plant growth, photosynthesis, and water use efficiency (Chaumont & Tyerman, 2014; Moshelion et al., 2015). As important regulators of the plant water balance, AQPs are excellent targets to increase our understanding of how plants can deal with drought stress and survive in arid environments (Shekoofa & Sinclair, 2018; Zargar et al., 2017; Zhang et al., 2019). Understanding the mechanisms that promote expression of AQPs would allow rapid identification of key AQP genes underpinning drought adaptation and provide a tool to screen natural populations to predict their abilities to cope with climate change. Such endeavors are especially important for natural habitats that are dominated by few foundational species, including the threatened sagebrush steppe ecosystem in western North America (Davies et al., 2011). Promoter sequence analyses, such as those in Lopez et al. (2013), may provide valuable information about what drives the expression of AQP genes for plant species in these habitats.

Researchers have primarily focused on studying the roles and importance of AQPs using crop and model plants; very little effort has been devoted to species occurring in natural environments. *Artemisia tridentata* Nutt. (sagebrush; Asteraceae) exists as a polyploid species complex with a history of hybridization (Freeman et al., 1991; McArthur et al., 1988; Taylor et al., 1964) that occupies environments with contrasting precipitation regimens and drought occurrences (Kolb & Sperry, 1999). This complex evolutionary history, in conjunction with limited resources available for research, has complicated genetic studies of this taxon. Natural variation in drought tolerance within this species may provide an excellent system to study the role of AQPs in plants’ responses to water stress and survival in arid environments (Maurel et al., 2010). This long‐lived shrub is the most ecologically important and dominant species of the steppes of northwestern North America (Karban, 2007; Leonard et al., 2000; Prevéy et al., 2010). Sagebrush steppe habitat once covered much of western North America (McArthur & Plummer, 1978; Mueggler & Stewart, 1980; Requena‐Mullor et al., 2019), though it is now threatened by various habitat disturbances (Barnard et al., 2019; Prevéy et al., 2010) and anthropogenic climate change (Richardson et al., 2017; Still & Richardson, 2015).

Anthropogenic climate change poses a great threat to many organisms across the globe. Average temperatures experienced an increase of 0.6°C within the 20^th^ century (reviewed in Jones et al., 2001), with a potential increase in future temperatures of 0.4°C per decade (IPCC, 2001). While shifts in species distributions have been identified across latitudinal and elevational gradients (Grabherr et al., 1994; Kullman, 2002; Lloyd & Fastie, 2003; Parmesan & Yohe, 2003; Penuelas & Boada, 2003; Sanz‐Elorza et al., 2003; Sturm et al., 2001; Walther, 2003; Walther et al., 2002), with ranges of many more species expected to shift (Bakkenes et al., 2002), the rate of climate change will likely outpace the ability of many species to acclimate, adapt, or disperse (Cang et al., 2016; Jezkova & Wiens, 2016; Quintero & Wiens, 2013; Wiens, 2016). Along with rising temperatures, many areas will see changes in precipitation and aridity, leading to increased water‐related stress in sessile plants (Gao & Giorgi, 2008; Zarch et al., 2017; reviewed in Huang et al., 2017).

Given the rapid improvements in genomic tools, use of draft genomes in the study of nonmodel organisms can greatly decrease cost and help generate focused genome‐to‐phenome (G2P) hypotheses for long‐term experiments (reviewed in Wojahn et al., 2021). A draft genome can be assembled and then mined for relevant genetic information, as opposed to a more classical G2P experiment, in which genetic/transcriptomic data would be linked to an experimentally induced phenome. In this study, we explored the evolution of AQP genes that may promote drought adaptation in plants using diploid *Artemisia tridentata* Nutt. subsp. *tridentata* (2*n* = 2*x* = 18; McArthur & Sanderson, 1999). *Artemisia tridentata* subsp. *tridentata* is a nonmodel plant that can be difficult to use in genetic research, with few resources currently available, though propagated lines are in development (Barron et al., 2020). Here, we test the hypothesis that the genome of the drought‐tolerant taxon *A. tridentata* subsp. *tridentata* will be enriched with AQP genes, particularly those of the *PIP* and *TIP* subfamilies, which produce proteins key in channeling water between cells, by being located in the plasma membrane, and the tonoplast, respectively, and that differentiation of promoter sequences have driven the evolution of novel functional groups of these AQP genes underpinning biochemical pathways adapted to drought tolerance.

These hypotheses were tested by assembling a draft genome of a diploid *A. tridentata* subsp. *tridentata* (2*n* = 2*x* = 18) and mining for AQP genes. Candidate AQP genes were characterized with regard to their amino acid sequences, predicted three‐dimensional structures, promoter elements, and phylogenetic inference. AQPs mined from the draft genome were compared to those of an annual, nondrought‐tolerant congener, *Artemisia annua* L. (Shen et al., 2018), to determine whether the genome of *A. tridentata* subsp. *tridentata* is enriched for AQP genes, which may confer increased tolerance to drought stress.

## 2 MATERIAL AND METHODS

### 2.1 Sampling, genome sequencing, and assembly

A diploid (2*n* = 2*x* = 18) individual of *Artemisia tridentata* Nutt. subsp. *tridentata* from a common garden in Orchard, Idaho (USA), grown from seed collected near Mountain Home, Idaho, USA (43.3371, −116.0081), was sampled for DNA extraction and genome sequencing (1C = 2.98 Gbp; Richardson et al., 2012; other individuals of this species have been found to have much larger genome sizes: 1C = 4.12–4.21 Gbp; Garcia et al., 2008). This individual, known as IDT2‐2, was grown as part of a long‐term experiment conducted by the USDA Forest Service in the Orchard common garden (Richardson & Chaney, 2018). DNA extraction was performed at Boise State University using a Qiagen Plant Mini kit per manufacturer protocol and quantified using Qubit (Thermo Fisher Scientific, Waltham, MA USA). A sample of 30 ng/μl was sent to the HudsonAlpha Institute for Biotechnology (Huntsville, AL, USA) for sequencing. A PCR‐free 2 ×150 bp paired‐end library of 350 bp standard was constructed using the Illumina TruSeq DNA PCR‐Free LT Library Preparation Kit (cat #20015962). After construction, the library was assessed for concentration by a Qubit™ fluorometer, fragment size with an Agilent Bioanalyzer, and optimal loading concentration by qPCR.

### 2.2 De novo genome assembly

A whole‐genome shotgun sequencing and standard sequencing protocols were utilized to sequence the *Artemisia tridentata* subsp. *tridentata* genome. Reads were generated using the Illumina NovaSeq platform at the HudsonAlpha Institute for Biotechnology in Huntsville, Alabama (USA). Two TruSeq PCR free 400 bp insert 2 × 150 Illumina fragment libraries (176.3 × raw coverage) were generated. Prior to assembly, Illumina fragment reads were screened for PhiX contamination. Reads composed of >95% simple sequence were removed. Illumina reads <50 bp after trimming for adapter and quality <20 were removed. The final read set consists of 5,388,578,188 reads for a total of 169.9× of high‐quality Illumina bases. The genome assembly was generated by assembling the 5,388,578,188 Illumina reads (169.9× sequence coverage) using the HipMCL assembler (version 1.1‐27‐g69eb6141; Azad et al., 2018). To validate our draft genome assembly for gene mining purposes, BUSCO V.5.1.2 (Seppey et al., 2019) was used to estimate the percent of orthologous genes from the eukaryote (*n* = 255) and eudicots (*n* = 2326) db10 databases that were completely and partially assembled or missing within the draft genome. BUSCO analysis was performed using default parameters and the Augustus gene prediction algorithm (Stanke & Waack, 2003).

### 2.3 Identification and validation of Aquaporin genes

Scaffolds containing candidate AQP genes were identified via BLASTN search using a custom reference BLAST database containing all DNA sequences available from GenBank encoding for AQP genes (for data regarding *Artemisia tridentata* subsp. *tridentata* AQP genes used in downstream analyses, refer to Table S1). Accession numbers of top BLAST hits were recorded, as well as positions of hits along scaffolds allowing the identification of genes that were fully contained within scaffold sequences. Open reading frames (ORFs) were predicted on scaffolds identified by the BLAST analysis using the *findORFs* function from the “ORFik” R package (Tieldnes & Labun, 2020). The number of predicted ORFs, strands, and positions along each scaffold were recorded. To identify ORFs coding for AQP codons, ORFs were converted into amino acid sequences and BLASTP analyses were run on the online BLAST portal. Top BLAST hits for each ORF coding for AQP exons were recorded to further refine gene hypotheses (Table S1).

### 2.4 Predicting secondary and tertiary protein structures

AQPs are characterized by having six helices, two loops, and the presence of NPA motifs in loops (Park et al., 2010). The online TMHMM server v. 2.0 (http://www.cbs.dtu.dk/services/TMHMM/) was used to predict the number of transmembrane helices (Krogh et al., 2001), and a custom R script using base R functions was used to predict number, positions and amino acid composition of NPA motifs, and number of amino acid residues (R Core Team, 2020; https://github.com/aemelton/DraftGenomeMineR). We confirmed locations of NPA motifs in loops by using output of the TMHMM analyses. These analyses were based on concatenated amino acid sequences resulting from the ORFs and BLAST analyses.

The Phyre2 online platform with default parameters (http://www.sbg.bio.ic.ac.uk/~phyre2/html/page.cgi?id=index) was used to predict and analyze AQP protein tertiary structures, functions, and locate amino acid regions where mutations would change functions (Kelley et al., 2016). This analysis was performed on AQP amino acid sequences with at least five predicted helices and two NPA motifs. Tertiary protein models were saved in pdb formats, and data on model accuracy and coverage, closest protein(s) in Phyre2 database, and locations of mutations along proteins were recorded to further validate and infer functions of AQP s in *Artemisia tridentata* subsp. *tridentata* (Table S1).

### 2.5 Phylogenetic analysis

The phylogenetic reconstruction based on amino acid sequences was used to (i) confirm the identities of putative AQP genes in *Artemisia tridentata* subsp. *tridentata* genome and (ii) compare them to those of *Artemisia annua* L. identified via the NCBI BLAST portal to identify potential candidates underpinning climate‐induced adaptations in *A. tridentata* subsp. *tridentata*, that may have evolved to function in potential new pathways relating to drought stress.

Extracted AQP amino acid sequences were aligned with AQP sequences from *Artemisia annua* and *Arabidopsis thaliana* (L.) Heynh. mined from GenBank. *Arabidopsis thaliana* AQPs were used as the initial queries for *Artemisia annua* mining from GenBank and for BLASTP identification hypotheses for *A. tridentata* subsp. *tridentata* due to high level of validation of these proteins (Quigley et al., 2001). The alignment was performed via the online mafft V.7 portal (https://mafft.cbrc.jp/alignment/server/; Katoh et al., 2019) using the E‐INS‐i algorithm. Phylogenetic reconstructions were performed via raxmlGUI 2.0.0‐beta.14 (Edler et al., 2020) using the protgamma model and 1000 rapid bootstrap replicates.

### 2.6 Promoter analysis

Promoter analyses were performed to investigate mechanisms triggering AQP gene expression using the approach described in Lopez et al. (2013). One and half kilobases of upstream sequence data were extracted from scaffolds containing at least 1500 bp upstream from the AQP start codon. Upstream sequences were analyzed for putative *cis*‐acting sequences (regulatory distal element; RDE) using the Plant *Cis*‐acting Regulatory DNA Elements (New PLACE) signal scan software package (https://www.dna.affrc.go.jp/PLACE/?action=newplace, Higo et al., 1999). These RDEs comprise short nucleotide motifs that occur upstream of the start codon of a gene and influence the expression of genes. Detected RDEs were sorted into biological categories which included light, abscisic acid (ABA), water stress, temperature stress, stress hormone, growth, and other stress, and targeted area of expression (e.g., leaf, aerial tissue, and roots) based on keywords for each RDE listed within the New Place database file using a custom R script (https://github.com/aemelton/DraftGenomeMineR). Due to some RDE’s having functions in multiple pathways, some RDEs could be categorized into multiple categories to account for their various functions (e.g., many drought‐responsive elements, such as DREDR1ATRD29AB, respond to both temperature and water stresses; Yamaguchi‐Shinozaki & Shinozaki, 1994). A Kruskal–Wallis test via the function *kruskal.test* in base R (R Core Team, 2020) followed by Dunn tests via the function *dunn.test* in the R package “dunn.test” V.1.3.5 (Dinno, 2017) were performed on category count data per gene to determine if there are differences in the occurrences of RDEs for any given category, indicating enrichment of RDEs in certain categories and indicating important triggers for the expression of these AQP genes.

A singular value decomposition (SVD) on the occurrence counts of RDEs for each category within upstream sequence for each gene was used to determine how AQPs may form functional groups, as proxy for biochemical pathways, with transcription potentially being promoted by similar processes. These analyses were performed using the “LinearAlgebra” and “Clustering” v.0.13.5 packages in Julia v.1.5.3 (Bezanson et al., 2017). Additionally, we performed a *k*‐means clustering analysis on the raw RDE category data to identify clusters of genes that have similar drivers of expression and presumably function in similar response pathways. To determine the most appropriate *k* for clustering, *k*‐means analyses were performed with *k* values ranging from three to 20 assessed by silhouette analysis using the “Clustering” package in Julia. The silhouette analysis aims to assess the cohesion of points within clusters and the separation of points from different clusters. This allows for the identification of the ideal number of clusters, *k*, by determining the *k* value that produces clusters with high cohesion and separation. Given that these RDEs play important roles in controlling expression, identifying clusters of AQPs based on the RDEs in principal component space (PC‐space) will allow us to potentially identify important drivers of the expression of these genes.

## 3 RESULTS

### 3.1 Draft genome assembly

In total, 3.33 million scaffolds containing3.82 million contigs were assembled. Contigs covered 97.87% of the total length of the scaffolds. These comprise 4.50 Gbp and 4.41 Gbp, for scaffolds and contigs, respectively, covering the entirety of the haploid genome (1C = 2.98 Gbp; Richardson et al., 2012). The N50 for all scaffolds and contigs were 324,094 and 409,868, respectively. The L50 for scaffolds and contigs were 3.5 and 2.6 kbp, respectively. Fifty‐nine scaffolds and 191 contigs greater than 50 kbp were assembled. BUSCO predicted 100 (39.2% of orthologues in database) and 1035 (44.5% of orthologues in database) complete orthologous genes from the eukaryote and eudicot databases, respectively. Eighty‐five (33.3% of orthologues in database) and 844 (36.3% of orthologues in database) orthologues were missing from the draft genome for the eukaryote and eudicot databases, respectively.

### 3.2 Identification and validation of Aquaporin genes

Eighty‐three scaffolds were identified as containing a total of 84 putative AQP genes. Of these, 50 scaffolds were predicted to contain fully assembled AQP sequences. Scaffold number 128070 contained two tandem, fully assembled putative AQP genes. Thus, a total of 51 AQPs were considered in downstream analyses (Table S1; File S1 contains scaffolds from which the AQP genes were extracted). Of these AQP genes, 11 were identified as *NIP*s, 21 as *PIP*s, three as *SIP*s, and 16 as *TIP*s based on BLASTP searches. Gene length ranged from 888 to 1658 nucleotides for the 51 AQP genes and included two to five predicted exons. Six variants of the NPA motif were found: NPA, NPS, NPV, NPT, PPA, and FPA. Relevant gene data (i.e., gene length, predicted exon number, and NPA motifs) are listed in Table S1.

### 3.3 Predicting secondary and tertiary protein structures

A total of 19 putative AQP genes met criteria for Phyre2 analyses. All genes were validated as members of the AQP gene family. Identification of TIP proteins using the Phyre2 database suggested these unidentified *TIP*s belong to the *TIP2‐1* group. Phyre2 predicted that the greatest effects of mutations would occur at the NPA motifs. Mutations expected to affect function were identified in two NIP5‐1 proteins, which have NPS and NPV motifs (Table S1).

### 3.4 Phylogenetic reconstruction

**FIGURE 1:**
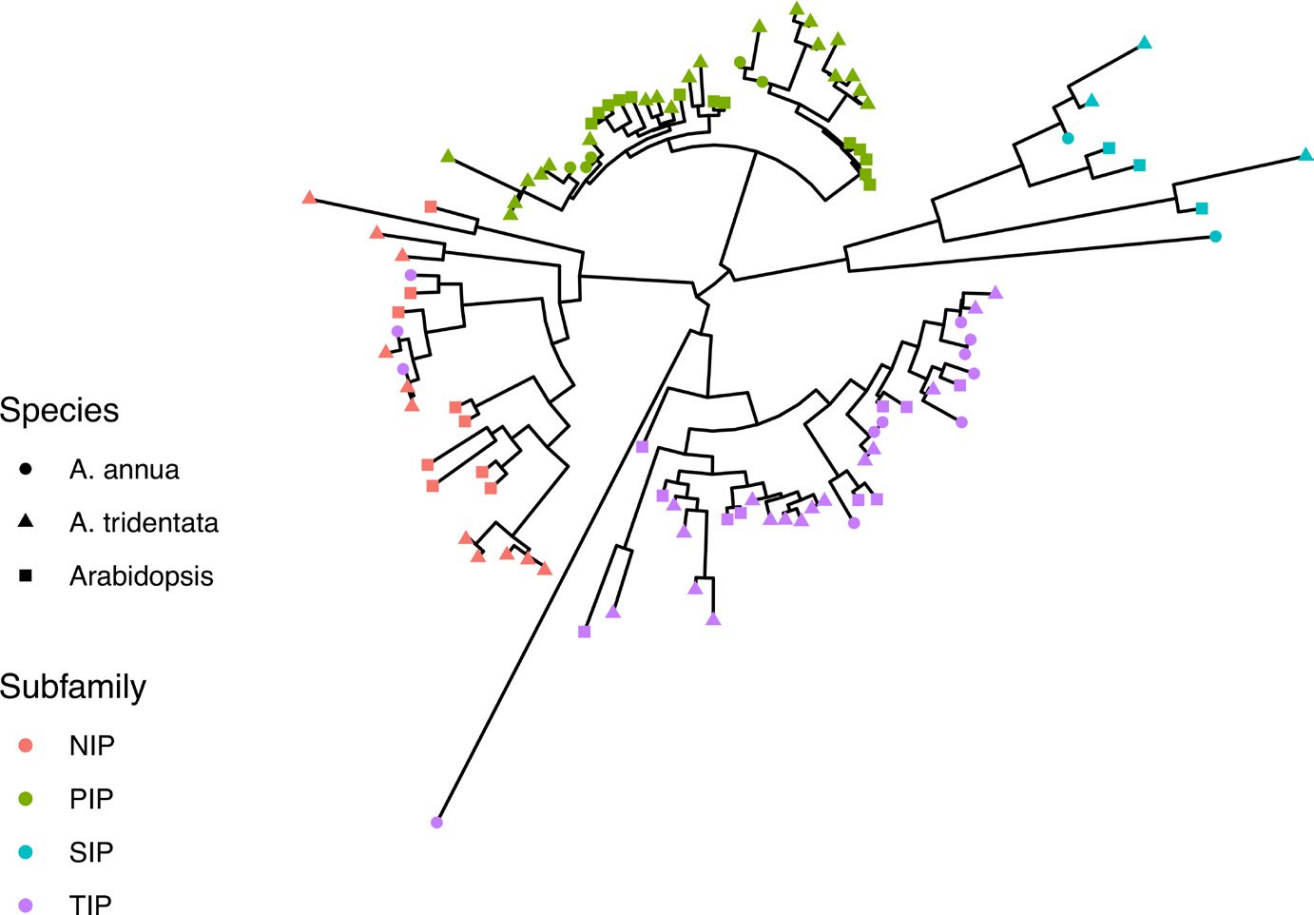
Midpoint rooted phylogeny of AQP genes from Arabidopsis, Artemisia annua, and Artemisia tridentata subsp. tridentata. Several genes previously identified as TIP genes in Artemisia annua via other methods formed a clade with the NIP genes, indicating these were previously misidentified and belong to the NIP subfamily

Phylogenetic reconstructions of AQP amino acid sequences from *Artemisia tridentata* subsp. *tridentata* (51 protein sequences), *A. annua* (19 protein sequences), and *Arabidopsis thaliana* (35 proteins sequences) recovered four clades, each comprising a given subfamily (Figure 1; File S2 contains aligned amino acid sequences used in this analysis). One exception was the *Artemisia annua* gene PWA34518_1. While originally identified as *NIP5‐1* by BLASTP analysis, PWA34518_1 formed a clade with members of the *SIP* subfamily. Several other *A. annua* AQPs that were not identified to the subfamily level were found to be members of the *TIP* subfamily within the phylogeny. Phylogenetic reconstructions for AQP genes revealed several potential duplication events for AQPs within *Artemisia tridentata* subsp. *tridentata*. These putative duplications were particularly common in the *PIP* and *TIP* subfamilies, with *PIP1‐3*, *PIP1‐4*, *PIP2‐2*, *PIP2‐4*, *TIP*, and *TIP2‐1* comprising multiple copies (Figure 1). Genes identified as *TIP* genes for *Artemisia tridentata* subsp. *tridentata* formed a clade with the *TIP2‐2* and *TIP2‐3* genes of *Arabidopsis thaliana*, suggesting these unidentified *TIP*s are *TIP2* genes. *TIP2‐1* genes formed a distinct clade sister to the clade containing *TIP2‐2* and *TIP2‐3* genes. Therefore, the *TIP* genes are most likely *TIP2‐2* or *TIP2‐3* homologs (Figure 1). Phylogenetic analysis revealed that a *PIP* gene identified via BLAST as a *PIP2‐1* gene is actually a *PIP2‐4*, as it forms a clade with the *PIP2‐4* and not *PIP2‐1* genes. The *Artemisia tridentata* subsp. *tridentata NIP1‐1* gene has also experienced numerous putative duplication events, comprising five AQP genes. (Figure 1; Table S1).

### 3.5 Promoter anal–ysis

Of the 51 AQP genes retained for further analysis, 29 were on scaffolds with at least 1.5 kbp upstream sequence to be included in promoter analyses (File S3 contains sequences extracted for this analysis). In total, 224 RDEs were identified across all upstream sequences. Light, ABA, and stress hormones, including cytokinin and auxin, were the most represented categories, with 420, 189, and 157 occurrences of classified RDEs in total across all scaffolds, respectively. The RDE “CACTFTPPCA1” was the most prominent RDE among these genes, with a total of 636 occurrences. Other highly prominent RDEs include the following: DOFCOREZM (593), CAATBOX1 (515), ARR1AT (423), and GATABOX (373).

Most RDEs play roles in tissue‐specific expression, particularly in the aerial tissue. The most prominent RDE, CACTFTPPCA1, promotes expression in leaf mesophyll. Some RDEs identified target expression in roots: NTBBF1ARROLB (56), OSE1ROOTNODULE (43), OSE2ROOTNODULE (105), and ROOTMOTIFTAPOX1 (263). These root‐specifying promoter elements were most prominent in the upstream regions of several *NIP* and *PIP* genes, particularly *NIP2‐1*, *PIP1‐3*, *PIP1‐4*, and *PIP2‐4*. One TIP gene was also found to be enriched for ROOTMOTIFTAPOX1.

Results of the Kruskal–Wallis and Dunn tests show that light category RDEs are statistically significantly enriched relative to all other categories (*p*‐values = .0002 and 0 for ABA and all other categories, respectively). ABA category RDEs were statistically significantly greater than all categories except light (*p*‐value = .0002) and stress hormone (nonstatistically significant difference), with p‐values of 0.0006 for growth, 0.0010 for other stress, and 0 for temperature and water stress. Growth category RDEs occurred statistically significantly less than light (*p*‐value = 0), ABA (*p*‐value = .0006), stress hormone (*p*‐value = .0114), temperature (*p*‐value = .0097), and water stress (*p*‐value = .0040) category RDEs. Other stress category RDEs occurred statistically significantly more than temperature (*p*‐value = .0061) and water stress (*p*‐value = .0024) category RDEs, and statistically significantly less than those of light, ABA, and stress hormone (*p*‐value = .0174) category RDEs. Stress hormone category RDEs occurred statically significantly more than temperature stress (*p*‐value = 0) and water stress (*p*‐value = 0) category RDEs. Temperature stress category RDEs did not occur statistically significantly more than RDEs of any other category.

Silhouette analysis results suggested a *k* of three was most appropriate to describe the clustering of gene promoter sequences in PC‐space. Clustering in PC‐space was not related to the AQP phylogeny; at least one member of each subfamily was present in each of the three clusters, with members of each gene group also being spread out in PC‐space and clusters.

**FIGURE 2:**
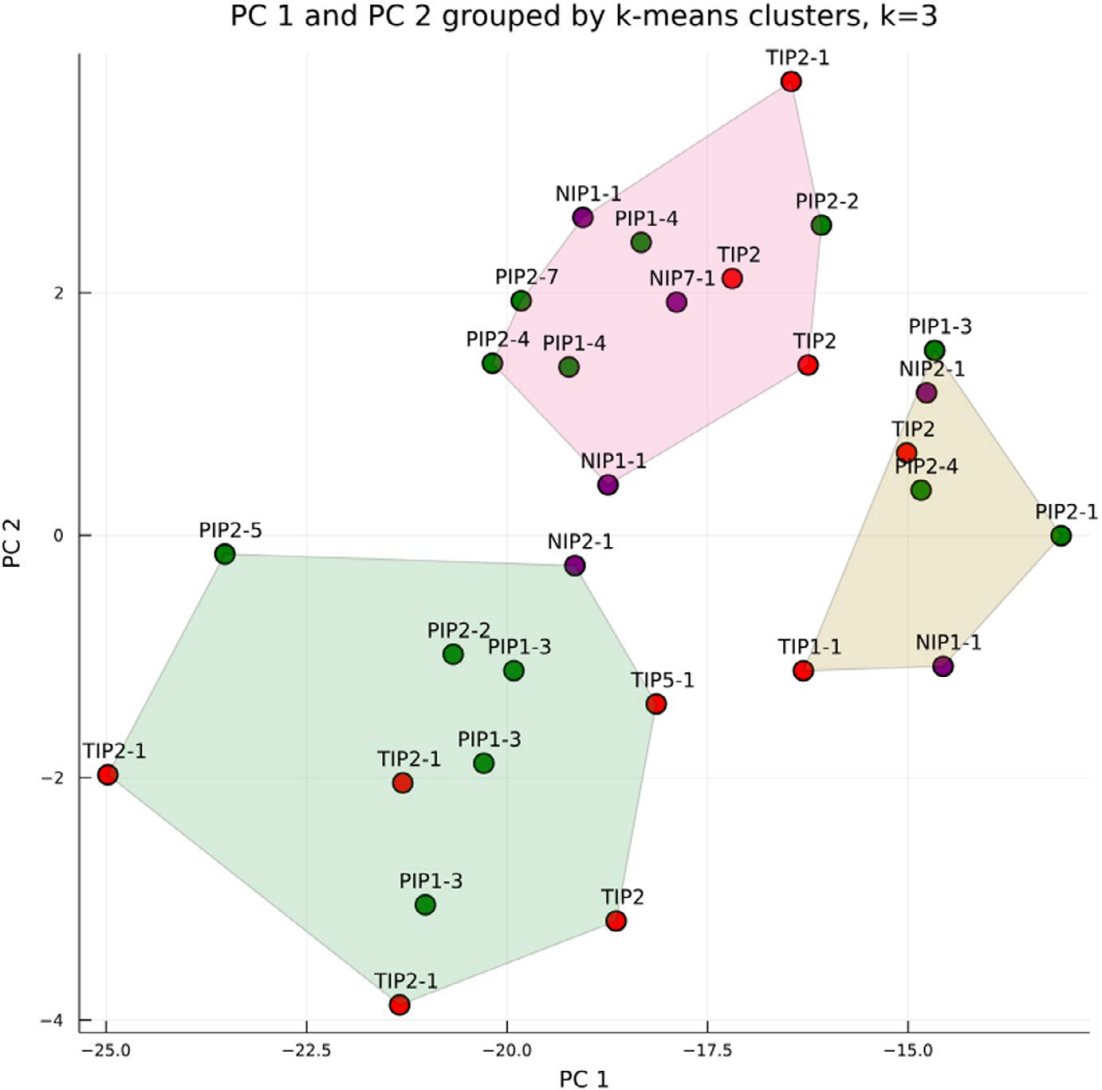
Result of SVD for RDE categories across AQP genes. Three distinct clusters were identified in the silhouette analysis, each enclosed in a convex hull. Each cluster consists of genes from multiple AQP subfamilies (NIP, purple; PIP, green; and TIP, red). PC1 explained 75% of variance, while PC2 explained 7.5% of variance

SVD of RDE categories (Figure 2) showed that light has the greatest influence on AQP expression (PC1 = 72% variation explained), while a combination of ABA and water stress were the secondary drivers of AQP expression (PC2 = 7.5% of variation explained). *PIP* genes were most greatly influenced by PC1, primarily light category RDEs, while *TIP*s were primarily driven by PC2, which included ABA and water stress category RDEs. *NIP* gene RDEs occupied an area of PC‐space, which was influenced by both PC1 and PC2. The most highly represented genes, *PIP1‐3* and *TIP2‐1*, each show at least one copy greatly diverging in PC‐space (Figure 2). Several copies of each of these two genes also occupy an area of PC‐space that indicates their expression is largely driven by ABA, water, and temperature stresses.

**FIGURE 3:**
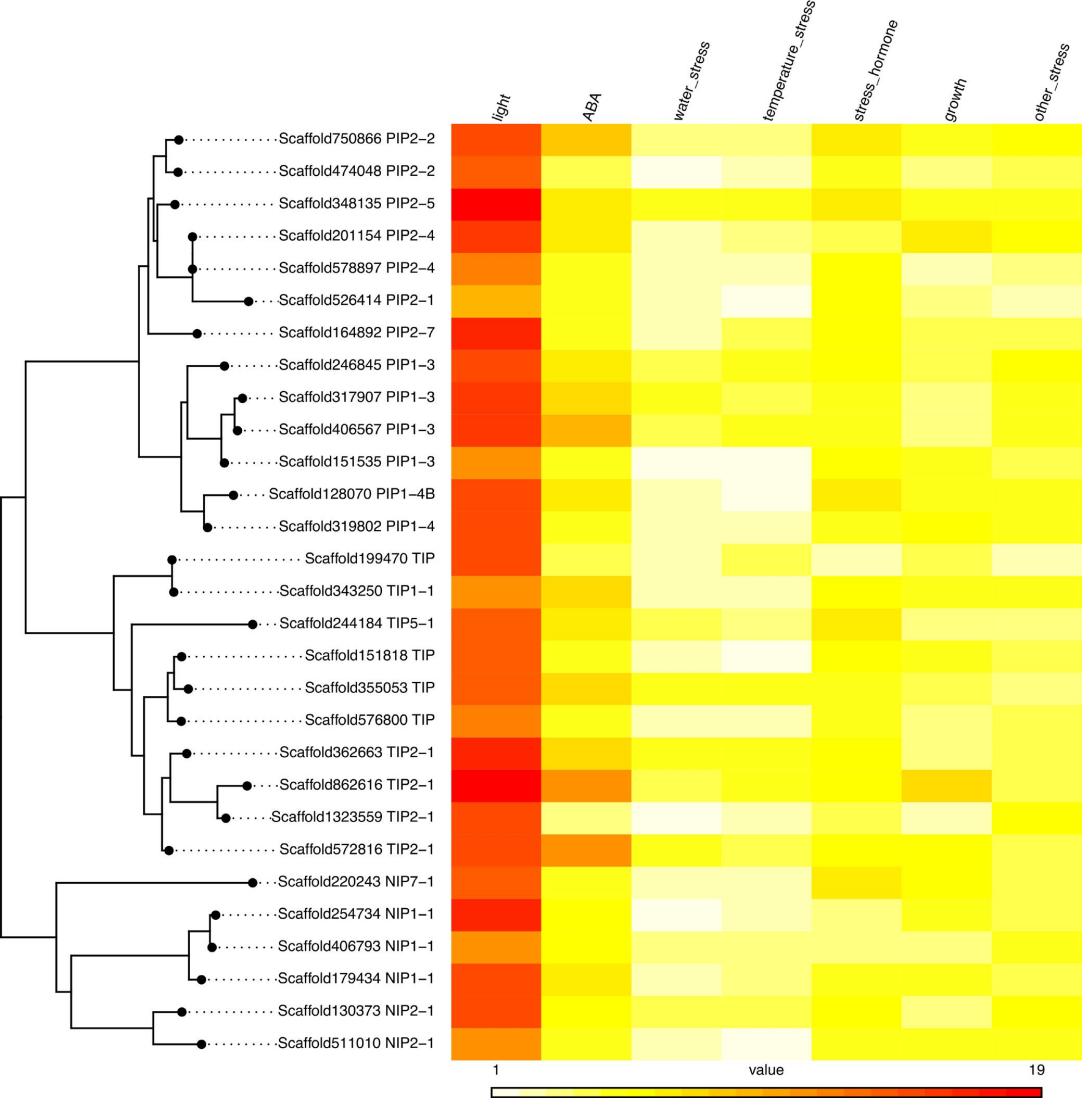
Heatmap of RDE categories per scaffold along with phylogenetic context for each gene. The value within each cell is equal to the number of occurrences of RDEs for a given category within the promoter sequences of each gene. Per Kruskal–Wallis and Dunn test results, the light category of RDEs was significantly enriched relative to all other categories. ABA category RDEs were significantly enriched relative to all other categories except light. Temperature category RDEs were the least prevalent and did not occur significantly more than those of other categories

Assessing RDE enrichment per AQP gene in a phylogenetic context revealed that putative AQP paralogues have experienced some degree of differentiation (Figure 3). For example, the two *PIP1‐4* genes included in promoter analyses differ in their enrichment for ABA, temperature stress, and growth categories. The *PIP2‐4* clade exhibits clear differentiation in their enrichment of RDE in all categories except water stress and stress hormones. *PIP1‐3* RDEs have primarily differentiated in the light and ABA categories. *TIP2‐1* genes have experience clear differentiation of RDEs in all categories except the stress hormone category.

## 4 DISCUSSION

### 4.1 Providing a draft genome to investigate drought tolerance

A first draft genome for *Artemisia tridentata*, a keystone species of western North America, was successfully assembled for use in genome mining and drought adaptation research. The individual used for this study was previously estimated to have a diploid genome size of 5.95 Gbp (6.09 pg; Richardson et al., 2012). Our draft genome assembly is at least four times that of *Artemisia annua* (1.38–1.78 Gb; Liu et al., 2018; Shen et al., 2018), while maintaining the same number of chromosomes (2*n* = 2*x* = 18). Draft genome assemblies for both species represent essentially complete haploid genomes (Shen et al., 2018). The draft genome assembled for *A. tridentata* subsp. *tridentata* was assembled without filtering of homologous scaffolds or assembling haploid genome sets. This does not affect the results of the analyses described here, as no homologous scaffolds containing AQPs were found. The BUSCO analysis demonstrated that this genome assembly was of sufficient quality to be mined for genes, as nearly two‐thirds of eudicot orthologues (63.7%) were partially or fully recovered. While sufficient for gene mining, genes were undoubtedly missed in our analyses due to the incontiguous nature of the draft assembly.

The methods described in this study provide a clear methodological pathway to validate genes within draft genome assemblies. While not all the 84 putative AQP genes were fully assembled within scaffolds and were thus excluded from downstream analyses, we were able to validate to a high degree the identity of 51 of the fully assembled putative AQPs. Given the results of the BUSCO analysis, it is likely that around 30% of AQP genes in the genome are left to be discovered. Our combination of BLAST, phylogenetic reconstruction, and prediction of tertiary structure allowed for high confidence validation of many of the putative *Artemisia tridentata* subsp. *tridentata* AQP genes (Figure 1; Table S1). While not all genes were validated using all methods, we can still be confident that they fall within the AQP gene family. Many of these genes were validated to at least subfamily and clade level (e.g., *PIP1‐1*; Table S1).

### 4.2 Enrichment of AQPs in a drought‐tolerant species

Our results indicate that the genome of drought‐tolerant *Artemisia tridentata* subsp. *tridentata* has at least 51 AQPs (Table S1). This number of AQPs is relatively high when compared to other species. Based on genome‐wide identification, Deshmukh et al. (2016) reported the number of AQPs in 31 species, ranging from 23 in the moss *Physcomitrella patens* to 72 in *Glycine max* (soybeans). Within the list presented in this review (Deshmukh et al., 2016), only four species have more AQPs than *A*. *tridentata* subsp. *tridentata*. Notably, this species has a much higher number of AQPs than its congener *A*. *annua*, which has 19 AQPs. This difference in gene enrichment could be due to whole‐genome duplication followed by diploidization and adaptation to more arid climates of western North America. The average genome size of *Artemisia* species in the North American group of the *Tridentatae* subgenus is approximately twice that of other *Artemisia* species (Garcia et al., 2008; Pellicer et al., 2010). The North American group of *Artemisia* subgenus *Tridendatae* likely diverged from the Asian clade approximately 10.8 mya (± 1.5 my; Sanz et al., 2011) after a vicariance event from Asia to North America via the Bering Land bridge. Given the divergence times, difference in genome sizes, and different climatic regimes occupied by the two species (Figure S1 highlights the difference in occupied climatic niches of these two species), it is likely that genomic processes, such as polyploidization followed by diploidization, and adaptation to a drought‐prone and arid environment have driven the evolution of the AQP gene family in the genome of *A*. *tridentata* subsp. *tridentata*.

The *PIP* and *TIP* subfamilies were the most enriched in the *Artemisia tridentata* subsp. *tridentata* genome, relative to the *A*. *annua* genome. Putative duplications of AQPs within the *Artemisia tridentata* subsp. *tridentata* genome seem to largely fall within the PIP and TIP subfamilies (Figure 1). These two subfamilies are the primary AQPs that function in water transport across the plasmalemma and tonoplast (Bienert et al., 2018; Siefritz et al., 2002; Song et al., 2016). The *PIP*s and *TIP*s identified in the *Artemisia tridentata* subsp. *tridentata* genome almost exclusively have NPA motifs, though one FPA and five PPA motifs were identified in proteins of the *PIP* subfamily (*PIP1‐3*, *PIP2‐1*, *PIP2‐2*; Table S1). The NPA to PPA mutation has been reported in *Linum usitatissimum*, though otherwise appears to be a rare change (Shivaraj et al., 2017). The conservation of these motifs is essential for the movement of water across membranes, as modifications of the NPA motifs lead to changes in the specificity, including nitrogenous compounds transported by *NIP*s. The asparagine residue in the NPA is highly conserved, as it provides helix cap stability and functions in cation exclusion (Wree et al., 2011). Therefore, it is likely that the AQPs with FPA and PPA motifs have experienced some degree of functional differentiation, though these have not been assessed. *NIP5‐1* contains NPS and NPV motifs that would likely alter the function of the gene, per Phyre2 analysis. While these are deviations from the typical NPA motif, AQP genes with variations of the NPA motif and confirmed function in water transport have been identified in other organisms (Ishibashi, 2006).

### 4.3 Light drives AQP expression

The most enriched RDE category presented here was “light,” occurring statistically significantly more than RDEs of any other category, with the GATABOX element being the most common. This RDE functions in chlorophyll a/b binding in aerial tissue. The observation of light‐responsive elements in *Artemisia tridentata* subsp. *tridentata* AQPs is consistent with several studies that have reported upregulation of AQPs in response to light (Baaziz et al., 2012; Cochard et al., 2007; Lopez et al., 2013). Moreover, these increases in AQPs expression were correlated to increases in leaf hydraulic conductivity, presumably adjusting water supply to transpirational demand (Baaziz et al., 2012; Lopez et al., 2013). However, light effects on aquaporin expression and leaf hydraulic conductivity vary between species. For example, *Juglans regia* showed a fourfold increase in hydraulic conductivity with light, while the increase was minimal upon illumination of *Salix alba* and *Quercus rubra* (Baaziz et al., 2012; Rockwell et al., 2011). Given the large complement of AQPs with light‐responsive elements in *Artemisia tridentata* subsp. *tridentata* (Figure 3), it would be interesting to determine how its hydraulic conductivity response to light compares to that of other species. ABA‐responsive elements and the MYCCONSENSUSAT RDE were also highly enriched. The latter has functions in both cold responses associated with *CBF/DREB1* genes and drought responses associated with dehydration‐responsive gene, *rd22*, genes (Liu et al., 2015). This is likely an important promoter element for *Artemisia tridentata* subsp. *tridentata*, which experiences long periods of drought during the summer, and cold to freezing temperatures during the winter (Kolb & Sperry, 1999; Lambrecht et al., 2007).

The presence of many tissue‐specific RDEs indicates that many of the identified AQPs are expressed primarily in aerial tissues. Since many RDEs in these AQPs have roles in light response, their expression in these tissues would be expected. Also, the RDE analysis suggests special expression in the leaf mesophyll, a tissue enriched in chloroplasts where photosynthesis occurs. *Artemisia tridentata* has a compact mesophyll, with air spaces mainly limited to the substomatal cavity (Downs & Black, 1999). Tight packing of cells may increase the proportion of transcellular water transport over the apoplastic pathway (Maurel & Prado, 2017). Under this scenario, high AQP expression in the mesophyll and other leaf parenchyma cells may be particularly important for regulating leaf water balance. Apart from water, AQPs in the mesophyll may facilitate the movement of other molecules across membranes, including CO_2,_ which could contribute to increase mesophyll conductance to this gas during photosynthesis (Carriquí et al., 2019; Flexas, Bota, et al., 2006; Flexas, Ribas‐Carbó, et al., 2006; Singh et al., 2020). Overall, the presence of leaf‐specific RDE in several of the identified *PIP*s and *TIP*s are congruent with observations made in other species showing high expression of these subfamilies in leaves (Heinen et al., 2009). We also found some *PIP*s and *NIP*s with promoter elements indicative of root localization. Unfortunately, no *SIP* genes had sufficient upstream sequence to meet criteria for inclusion in promoter analyses.

### 4.4 Differentiation of the drivers of AQP expression

Comparative analyses provided evidence for RDE content differentiation among homologs of some AQP genes, particularly *PIP1‐3* and *TIP2‐1*. These genes are most highly represented in the RDE analyses, with four copies each, and each had at least one copy that took on novel functions relative to the other copies. Copies of these genes, and genes with multiple copies, do not occupy the same PC‐space as members of their homologs (Figure 2). In some cases, a copy of a gene may occupy quite distant space from other gene copies. This indicates that promoter sequence differentiation, to varying degrees, has occurred in these genes. Given the results of SVD and phylogenetic comparisons, the *PIP1‐3* and *TIP2‐1* genes appear to be strong candidates for future research as drought tolerance genes in *A. tridentata* subsp. *tridentata*. Both genes occur in multiple copies within the genome, and they have experienced RDE category differentiation (Figures 1, 2, 3). These genes also have copies that have diverged in promoter sequences to likely play a greater role in ABA and drought stress pathways (Figure 2). So, while these genes may share similar amino acid sequences with their respective homologs, they do exhibit differentiation RDE composition, suggesting differences in biological pathways that would drive their expression.

### 4.5 Perspectives

Overall, this research provides genomic resources and valuable hypotheses for further work on *Artemisia tridentata* subsp. *tridentata*. While our methods do not replace larger G2P experiments, they do offer a more rapid and cheaper method to acquire and analyze data that will generate testable G2P hypotheses, which can lead to more focused and efficient experiments. We will be able to test such hypotheses thanks to the development of a novel in vitro method of propagation developed specifically for *A‐ tridentata* subsp. *tridentata* (Barron et al., 2020). We are currently in the process of generating several individual lines, which will be used in generating more higher quality draft genomes, assembling a phased diploid genome for this taxon, and to conduct genotype‐by‐environment experiments.

## 5 CONCLUSIONS

We see clear evidence that the genome of the drought‐tolerant taxon, *Artemisia tridentata* subsp. *tridentata*, contains far more AQP genes, particularly *PIP*s and *TIP*s, than the genome of a nondrought‐tolerant congeneric species, *A. annua*. This is likely due to *in situ* processes within the genomes of *Artemisia tridentata* and the North American *Artemisia* clade. This research has also led to important hypotheses generation for future research, particularly that *PIP1‐3* and *TIP2‐1* genes could potentially confer increased drought adaptation in this foundational, keystone species.

## CONFLICT OF INTEREST

The authors have no conflicts of interest to disclose.

## AUTHOR CONTRIBUTIONS

**Anthony E. Melton:** Conceptualization (equal); Data curation (equal); Formal analysis (lead); Investigation (lead); Methodology (equal); Validation (lead); Visualization (lead); Writing‐original draft (lead); Writing‐review & editing (equal). **James D. Beck:** Formal analysis (supporting); Writing‐review & editing (supporting). **Stephanie J. Galla:** Visualization (supporting); Writing‐review & editing (supporting). **Jerry W. Jenkins:** Data curation (supporting). **Lori Handley:** Data curation (supporting). **Min Kim:** Data curation (supporting). **Jane Grimwood:** Data curation (supporting). **Jeremy Schmutz:** Data curation (supporting). **Bryce A. Richardson:** Data curation (supporting); Writing‐review & editing (supporting). **Marcelo Serpe:** Formal analysis (supporting); Methodology (supporting); Writing‐review & editing (supporting). **Stephen Novak:** Methodology (supporting); Writing‐review & editing (supporting). **Sven Buerki:** Conceptualization (equal); Data curation (equal); Formal analysis (equal); Funding acquisition (lead); Investigation (equal); Methodology (equal); Project administration (equal); Writing‐review & editing (equal).

## Supporting information

Fig S1

File S1

File S2

File S3

Table S1

Supplementary Material

## Data Availability

Scripts used in this research have been deposited at https://github.com/aemelton/DraftGenomeMineR. The raw reads (accessions SRR14309371, SRR14309372, and SRR14309373) and draft genome assembly (accession JAHAUY000000000) have been submitted to NCBI as SAMN18747788 under PRJNA722258. Supplementary documents, including scaffolds and alignment fasta, have been submitted as part of the SI.
