# Supplementary material for: A draft genome provides hypotheses on drought tolerance in a keystone plant species in Western North America threatened by climate change
Source: Ecol Evol. 2021 Oct 19;11(21):15417–29. doi: 10.1002/ece3.8245 (PMC8571618; doi:10.1002/ece3.8245)
Supplement: Supplementary file 6 — Supplementary Material [file ECE3-11-15417-s004.docx]

**Supplemental Information for:**

**Reversing the Genome-to-Phenome Research Pipeline: A Draft Genome Provides Hypotheses on Drought Tolerance in A Keystone Species Threatened By Climate Change**

Anthony E. Melton, Jim Beck, Stephanie J. Galla, Jerry Jenkins, Lori Handley, Min Kim, Jane Grimwood, Jeremy Schmutz, Bryce A. Richardson, Marcelo Serpe, Stephen Novak, Sven Buerki

**Figure S1.** Occupied climatic niches of (A) *Artemisia tridentata* subsp. *tridentata* and (B) *A. annua*. This figure was generated using occurrence data from GBIF (www.gbif.org/) and iDigBio (www.idigbio.org/), climate data from WorldClim (www. worldclim.org/), and a R code modified from a script by O. Broenniman available at <https://www.unil.ch/ecospat/en/home/menuguid/ecospat-resources/tools.html> (modified script available at [www.github.com/aemelton](http://www.github.com/aemelton)). These two species occupy distinct climatic niches, with little overlap (designated by purple). The greatest occurrence density in niche space is designated by darker cells in each respective plot (*A. tridentata* subsp. *tridentata* is red, while *A. annua* is green).

**Table S1** Information regarding the 51 AQP genes used in the analyses described in this paper. This information include length, gene hypotheses in validation steps, top BLAST hits, position in scaffold, ORFs identified, length of protein, and NPA motifs.

**File S1** Scaffolds extracted from draft genome that contain AQP genes used in downstream analyses.

**File S2** Aligned translated AQP sequences from *Arabidopsis thaliana*, *Artemisia annua*, and *Artemisia tridentata* subsp. *tridentata* used in RAxML phylogenetic reconstruction.

**File S3** Upstream sequences of at least 1.5kb extracted for use in promoter element analyses.
