# Supplementary material for: A draft genome provides hypotheses on drought tolerance in a keystone plant species in Western North America threatened by climate change
Source: Ecol Evol. 2021 Oct 19;11(21):15417–29. doi: 10.1002/ece3.8245 (PMC8571618; doi:10.1002/ece3.8245)
Supplement: Supplementary file 1 — Fig S1 [file ECE3-11-15417-s005.pdf]

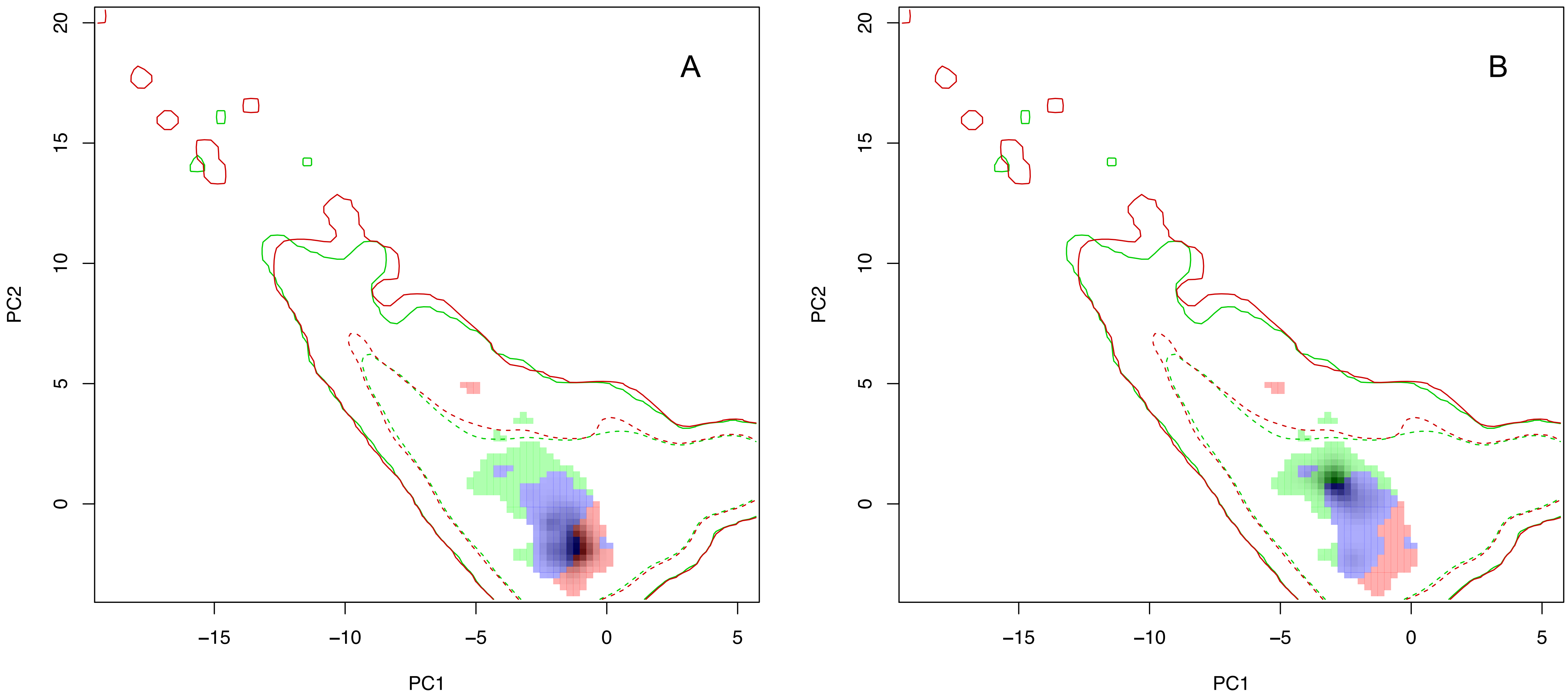

A
B
PC1
PC2
−15
−10
−5
0
5
10
15
20
